# An online survey of dietary quality during complementary feeding; associations with maternal feeding self-efficacy and adherence to dietary recommendations

**DOI:** 10.1186/s40795-022-00595-8

**Published:** 2022-09-09

**Authors:** Eleni Spyreli, Michelle C. McKinley, Moira Dean

**Affiliations:** 1 Centre for Public Health, Institute of Clinical Science Block A, Queen’s University Belfast, Grosvenor Road, Belfast, BT12 6BJ United Kingdom; 2 Institute for Global Food Security, Queen’s University Belfast, Belfast, BT9 5DL UK

**Keywords:** Dietary quality, Complementary feeding, Parental feeding practices

## Abstract

**Background:**

Parents are the gatekeepers of nutrition in early life and their feeding practices form children’s dietary behaviours. Although maternal characteristics have been associated with certain feeding practices, their relationship with overall quality of complementary feeding diets has not been explored. This study aimed to: assess dietary quality in complementary feeding age; explore its association with maternal and child characteristics; and evaluate the association between complementary feeding practices and child weight.

**Methods:**

An online cross-sectional survey captured data from a self-selected sample of mothers living in the UK with a healthy full-term child in complementary feeding age. A total of 466 mothers completed a questionnaire on their complementary feeding practices, demographics, anthropometrics, rates of maternal food neophobia, feeding self-efficacy, social support, postnatal depression and infant temperament. Dietary quality was assessed using the Complementary Feeding Utility Index (CFUI). Children were classified into underweight, normal weight, overweight and obese.

**Results:**

Majority of participants reported high levels of dietary quality as determined by a mean CFUI score of 80%. High dietary quality was associated with reliance on the NHS recommendation on timing of complementary feeding and maternal self-efficacy in promoting a healthy diet and limiting non-recommended foods. Responsive feeding, longer breastfeeding duration, frequent exposure to fruits and to a high variety of protein-rich animal foods were significantly associated with lighter child weight status. Consumption of sweetened drinks and delayed introduction of lumpy foods were associated with heavier child weight status.

**Conclusions:**

This study provided an evaluation of dietary quality in complementary feeding in a UK sample of children and explored its relationship with maternal and child attributes. Increasing understanding of the current complementary feeding recommendations and strengthening maternal feeding self-efficacy may be key for healthcare professionals and researchers to improving complementary feeding practices.

**Supplementary Information:**

The online version contains supplementary material available at 10.1186/s40795-022-00595-8.

## Background

Parents and caregivers are the sole providers of children’s nutrition in first years of life and their feeding practices can contribute to the development of healthy eating behaviours [1, 2]. To ensure a balanced nutrition during complementary feeding, diet should be characterised by a variety of nutrient-dense foods, by avoidance of non-recommended ingredients (e.g. salt, sugar) and by the gradual introduction of various textures (e.g. lumps) that will facilitate food acceptance [3, 4]. However, the 2011 Diet and Nutrition Survey of Infants and Young Children (DNSIYC), a dietary assessment of 2,683 children aged between 4 and 18 months in the United Kingdom, revealed parental feeding behaviours that compromise nutritional diversity and might give rise to excessive weight gain [5]. Results highlight frequent consumption of energy-dense and nutrient-poor foods and low variety of protein- and iron-rich foods. They also showed that most children consume more calories than they require. Initiatives that will develop a deeper understanding of how parents shape their feeding practices are required in order to best address non-recommended feeding practices and increase adherence to the complementary feeding guidelines.

In recent years, a growing body of evidence exists in relation to parental and infant predictors of feeding practices. Caregiver-child meal interactions are bidirectional and parents adapt their feeding behaviours in response to infant characteristics, such as temperament [6]. Infant temperament has been previously linked to infant feeding practices and specifically, the timing of complementary feeding [7–9].

Self-efficacy is another important aspect of parenting; it refers to parents’ self-judgement of own ability to perform a specific childcare-related task [10, 11]. Parental feeding self-efficacy, i.e. parents’ self-competence in feeding across a variety of situations, has been explored as a predictor of feeding decisions predominately among older children (> 2 years) [12–14]. In these explorations, which used distinct measures of feeding self-efficacy from one another, participants were invited to answer a number of items (on a Likert scale format) that reflect children’s dietary habits and to evaluate their ability to influence each of these habits. The relationship between maternal feeding self-efficacy and dietary intake during complementary feeding is explored to a lesser extent. A cross-sectional study that looked at the dietary intake of 1-year olds living in Australia reported that maternal feeding self-efficacy was positively associated with child vegetable intake and negatively associated with sweet snack/drink intake [15]. Further cross-sectional data point towards a direct relationship between maternal feeding self-efficacy and infant vegetable intake [16]. Therefore, the evidence in the area, albeit limited, provide a rationale for studying maternal feeding self-efficacy as a possible predictor of feeding practices during the complementary feeding age.

Evidence support that children’s food preferences can resemble those of parents [17]. Even though the association between children’s and parents’ food preferences is reported to be stronger when in school age and adolescence [18–20], it is possible that mothers’ rejection of unknown foods, can influence children’s intake by restricting access to the disliked foods. Previous qualitative work on food exposure and preferences during complementary feeding has highlighted that mothers’ reluctance to try unfamiliar foods can have a negative impact on the food selection offered to an infant [21]. Hence, by shaping a food environment lacking certain foods, mothers may restrict infant’s food intake.

Other mother-related characteristics that have been associated with infant feeding outcomes are postnatal depression and perceived social support [22–24]. Both mothers’ perceptions of low support from their male partner and depressive symptomatology have been shown to predict shorter breastfeeding duration and earlier introduction of complementary foods.

Previous research on parent-related factors have mainly focused on individual feeding behaviours e.g. timing of solid food introduction or fruit and vegetable consumption. In the last years however, nutritional assessment has shifted from assessing specific dietary constituents towards characterising quality of the overall diet [25, 26]. Numerous indices have emerged to assess dietary quality, which involves measuring how closely dietary intake aligns with nutritional recommendations and/or how varied the choices are across core food groups [26]. While there are numerous available tools to assess dietary quality in adults [27–29], there is only a limited number of studies aimed to characterise diet in early years and quantify its quality by assigning a score [30–34]. One of the diet quality assessment tools developed is the Complementary Feeding Utility Index (CFUI), designed using data on dietary intake from the Avon Longitudinal Study of Parents and Children cohort (ALSPAC) in order to determine the degree of adherence to complementary feeding guidelines in the UK, Australia, New Zealand and North America [34, 35]. The use of CFUI demonstrated high convergent validity with scores that were significantly associated with nutrient intake in infancy, as well as dietary patterns at 3 years.

While the maternal and child characteristics discussed above have been associated with individual infant feeding practices, their relationship with the overall diet remains unknown. 

This study aims:

1. to assess the dietary quality in a UK-wide sample of children in complementary feeding age by assessing the adherence of maternal feeding practices to the complementary feeding guidelines using a composite nutritional index.
2. to estimate the strength of the relationship between child dietary quality and a number of parent- and child-related characteristics (parental food neophobia, parental feeding self-efficacy, parental social support, postnatal depression, infant temperament).
3. to evaluate the association between complementary feeding practices and child weight status.
4. Findings of this study will help identify characteristics of mothers who are likely to show low adherence to the complementary feeding recommendations and will help develop targeted strategies to improve it.

## Methods

The study employed a cross-sectional survey design. Ethical approval was obtained from the Ethics Committee of School of Biological Sciences, Queen’s University Belfast. All participants gave informed consent by clicking on a mandatory field at the beginning of the survey.

### Participant selection

**Fig. 1:**
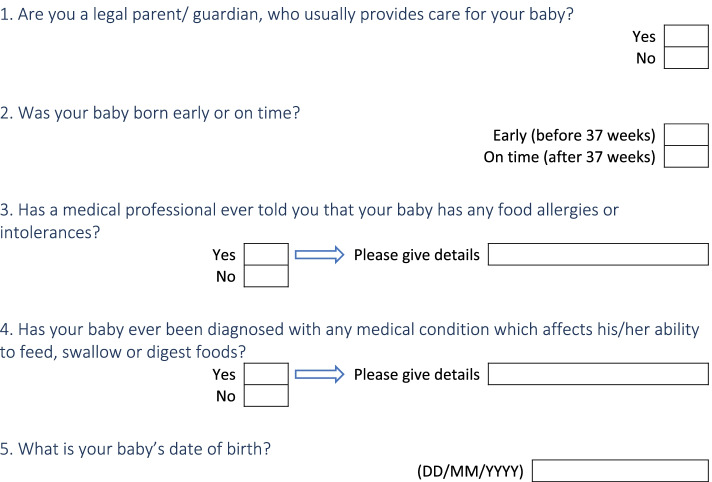
Questions that determined eligibility as these were displayed in the online survey

Participation was open to all parents who lived in the UK with a child that had been introduced to solid foods. The eligible child age range was based on the World Health Organisation definition of complementary feeding which takes place until 18–24 months. To increase parents’ ability to accurately recall early complementary feeding stages (i.e. introduction of solids and transition to lumpy textures), authors chose the lower end of the age range and decided that eligible children would be up to the age of 18 months at the time the survey was launched. Pre-term children or those with health issues (i.e. allergies/intolerances and conditions affecting the ability to feed) were excluded, as their nutritional experiences were distinct from infants with no such conditions. Eligibility was assessed after consent was obtained through five questions that confirmed that inclusion criteria are met (Fig. 1). Non-eligible individuals were unable to continue with the survey.

Authors engaged with a number of parenting support organisations and online forums across the UK and requested help with disseminating the call for participation in the study. The administrators of the groups that agreed to advertise the study posted the study information, the contact details of the research team and link to the online survey (see Additional file 1). Among others, the study information stated that the respondent was expected to be the person that usually feeds and cares for the child. A gift card worth £25 for ten randomly selected participants was used as an incentive for participation.

### Data collection

The questionnaire was piloted with five researchers (two males and three females) and two mothers for timing and clarity respectively. After feedback on wording and flow, minor adaptations were made. The survey was created on the online platform *Qualtrics* (Qualtrics XM 2019). It was launched online on the 17 May 2019 and remained open for completion for 3 months. Participation was anonymous and contact details submitted to enter the prize draw were stored separately from the survey data. The final questionnaire took approximately 20 min to complete and included questions in relation to the following characteristics and measures.

#### Dependent variables

1. Dietary quality. A questionnaire based on the one used in the ALSPAC longitudinal study [35] collected information on current complementary feeding practices. Certain questions included in the initial ALSPAC questionnaire were omitted and others were adapted to reflect current infant foods in the market. Data collected through the complementary feeding questionnaire were used to calculate a score for every component of the Complementary Feeding Utility Index (CFUI). CFUI, as detailed by Golley et al., consists of 14 components each one of which reflects one specific dietary recommendation. However, only 13 of its initial components were used for this study, as the item on iron-rich cereal products was not used. The reason for this was the variety of baby cereal products in the UK market (some are fortified with iron and some are not), which meant that an accurate estimation of iron rich cereal intake would require from mothers to state specific brands and cereal products used. Hence, estimating the frequency of intake of such products was deemed not feasible through the questionnaire (Table 1).

**Table 1:** Components of the Complementary Feeding Utility Index (CFUI)

Index item	Variable/s used to derive item
1.Breastfeeding duration	age in months when infant stopped breast feeding
2.Timing of introduction of solid foods	age in months when infant was fed foods or liquids except baby milk
3.Exposure to vegetables	consumption of vegetables prepared at home in times/day
4.Exposure to fruits	consumption of fruit prepared at home in times/day
5.Variety in protein foods	consumption of animal protein foods (meat, fish, eggs) in times/week
6.Exposure to sweet drinks^1^	types of drinks infant consumes per week
7.Exposure to confectionary and savoury snacks^2^	types of confectionary and unhealthy snack foods infant consumes per week
8.Timing of cow’s milk introduction	age in months when infant had cow’s milk as main drink
9.Exposure to tea and coffee	infant had tea or coffee (yes/no)
10.Exposure to commercial infant foods	age in months when infant introduced to meals with lumps
11.Timing of introduction to lumpy foods	consumption of ready-prepared meals in times/week
12.Number of daily meals and snacks	number of meals and snacks infant consumes per day
13.Feeding on demand	infant fed on demand or on schedule

^1^ Five types of sweetened drinks: caffeine-containing fizzy drinks, other sugar-sweetened fizzy drinks, sugar-free fizzy drinks, fruit drinks (including squash) and fruit juices ^2^ Four types of high salt, sugar and fat foods: chocolate, sweets, crisps and other savoury snacks

Each CFUI component obtains a score between 1 and 0; with 1 indicating full adherence to the specific recommendation, 0 very poor adherence and any values between 0 and 1 indicating moderate adherence. Overall dietary quality was determined as the overall CFUI score which was calculated by obtaining the mean of the 13 item scores and presented the same range (i.e. 0 being indicative of poor diet quality and 1 ideal). Individual component scores and overall index scores were calculated based on the methodology described by Golley and colleagues [34].

2. Child weight status. Participants provided information about their child’s most recent weight measurement before the survey. Children were then classified into categories: underweight, normal weight, overweight and obese. In the classification process, the research team used the growth charts for 0–4 years by the Royal College of Paediatrics and Child Health (RCPCH) [36], which are based on the WHO growth standards and are used by healthcare professionals in the UK when assessing child growth. Each child’s measurements were plotted on the appropriate Weight-for-Age growth chart for children’s age and gender, to identify the centile they were on. Being on the 91st centile or between 91st and 98^th^ classified as being overweight, and 98th and above obesity. On the other hand, measurements plotted from the 2nd centile and below were classified as underweight, as per the RCPCH guidance. Children whose measurements were in between were considered healthy weight. The strength of association between child weight status and maternal feeding practices was investigated.

#### Covariates

A number of covariates which were identified from the literature as potential contributors to quality of infant diets were measured. These were:

1. Food neophobia. Parents’ desire for a diet with variety was measured by using the Food Neophobia Scale (FNS). FNS is an instrument primary developed to measure willingness to try new and unfamiliar foods [37] and it has been widely used in UK adult populations [38, 39]. It is a self-administrated, 10-item questionnaire and high scores indicate low willingness to try novel foods. The score is obtained by summing the individual item scores measured on a 5-point Likert scale (ranging from strongly disagree to strongly agree) ranging from 0–50.

2. Parental self-efficacy in feeding. Detailed by Campbell and colleagues, this measure consists of two subscales: *Parental self-efficacy for promoting healthy eating* (with 4 items related to encouraging child’s sufficient intake of fruit, sufficient intake of vegetables, sufficient amount of food and dietary variety); and *Parental self-efficacy for limiting non-core foods* (with 3 items related to restricting child’s consumption of confectionary and sweets, crisps and savoury snacks, and sugar-sweetened drinks) [15]. Confidence for every behaviour/item was rated by participants on a 5-point Likert scale (‘not at all confident’ to ‘extremely confident’). An average score ranging from 0–5 was retrieved for every subscale.

3. Social Support. The Multidimensional Scale of Perceived Social Support (MSPSS) investigated social network support [40] and has been previously used in research with mothers living in the UK [41]. It is a 12-item instrument which addresses three sources of general support: friends, family and significant other (4 items for each source). Participants expressed level of agreement with each item-statement on a 5-point Likert scale. Total scores were retrieved by obtaining the mean and range from 0–5; higher total scores demonstrate perceptions of greater social support from these three sources.

4. Postnatal depression. The Edinburgh Postnatal Depression Scale (EPDS), a 10‐item self‐report scale designed to detect postnatal depression was used [42]. Developed in a UK sample of post-partum women, EPDS is quick, with a simple method of scoring (i.e. the overall score of the 10items, each one of which is scored on a 4-point scale) and is widely used to screen for depression even in mothers with older children [43]. The score ranges from 0 to 30 and higher scores indicate greater risk of depressive illness.

5. Infant temperament. The validated Infant Behaviour Questionnaire Revised (IBQ-R) Short Form was used to assess different domains of infant temperament. Specifically, caregivers are asked to report, on a 5-point scale, the relative frequency of occurrence of specified infant reactions across a broad array of contexts during the previous week [44]. IBQ-R explores 14 domains; including all of them would make the survey very lengthy and may have compromised the number of participants completing the survey. Instead, only three domains were used for this study i.e. “Activity Level”, “Distress to Limitations” and “Smiling and Laughter” (see Supplementary Table). Even though the use of these three domains alone has not been validated, they have formerly been associated with infant feeding practices in previous research [7, 9]. A mean frequency score was calculated for each one of the three domains ranging from 0–5, as per guidance of the authors of IBQ [44].

#### Additional information: Demographics, anthropometrics, prompts for introducing solid foods

The questionnaire also included demographics, such as parental and child age, gender, ethnicity, location of residence, marital status, educational attainment, number of children and annual household income. In this study household income was used as an indicator for socioeconomic status. Additionally, self-reported parental body weight and height were collected. Parental Body Mass Index (BMI) was then calculated and assigned to a BMI category based on the WHO definition [45]. Further data were collected regarding the cues that prompted participants to introduce solid foods; the choices given to participants to answer this question were informed by authors’ review of previous qualitative work in the area [46].

### Statistical analysis

Multivariate linear regression was utilised to identify associations between covariates and dietary quality (dependent variable), expressed as CFUI score on a continuous scale. Due to paucity of research regarding predictors of dietary quality in complementary feeding, all demographic variables and maternal BMI were adjusted for in the regression model. Influential factors expressed as categorical variables with more than 2 responses were separately investigated further for associations with dietary quality by creating dummy variables and incorporating them in the regression model by using one of them as a reference.

Contrary to the overall CFUI score, which was measured on a continuous scale, individual CFUI components take only specific values between 0 and 1. Therefore, the analysis performed to identify significant explanatory variables for each CFUI item was multivariate ordinal regression. The model adjusted for the same covariates as the linear regression model for dietary quality.

Multivariate ordinal regression was performed with children’s weight status as the dependent variable and a number of maternal feeding practices as potential predictors. The choice of confounding factors was based on previous literature that has linked maternal education, family income and maternal weight with feeding practices that in turn have been linked to overweight in early years [47]. The model was also adjusted for child’s age to account for any differences in feeding patterns due to the developmental stage children were going through at the time of the survey. For the independent variables that were measured on an ordinal scale, the lower categories were compared with the highest to produce the regression coefficients and P values.

All analyses were performed with IBM SPSS Statistics software (Version 25.0). Confidence intervals were set at 95% and statistical significance was established for *P* < 0.05. R square values were used as indicators of the proportion of variance in the feeding practices explained by the selected covariates (Nagelkerke R^2^ for ordinal regression and Adjusted R^2^ for linear regression).

## Results

### Sample descriptives

**Fig. 2:**
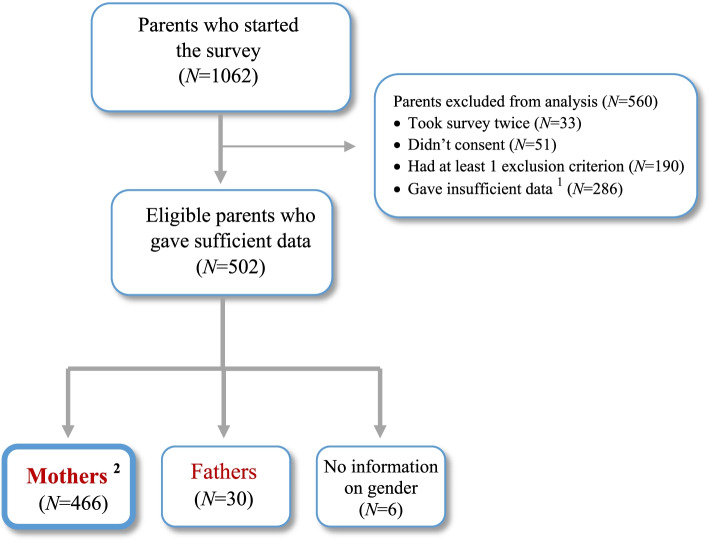
Numbers of excluded participants and those included in the final analysis

A total of 1062 parents started the online survey. Following exclusion of datasets of participants who did not meet the inclusion criteria and provided insufficient information, data from 466 mothers were included in the analysis. Partially completed questionnaires were included in the analysis, if the total CFUI score could be calculated from the responses. Comparison between mothers who gave sufficient information and those who did not, revealed no demographic differences. Both mothers and fathers participated in the survey. However, the current paper presents the results of the analysis of the mothers’ data only; the findings of the male sample have been published separately [48]. A flowchart with the numbers of parents included and excluded in the final analysis is presented in Fig. 2.

Descriptive statistics were presented in means and standard deviations (SD) for continuous variables, and proportions (%) for categorical. The majority of mothers in the sample were white (97%), had attended tertiary education (76%), had an annual household income of 40-60 K pounds (57%) and had one child (70%) (see Table 2). Their average age was 31 years and the mean age of children was 11 months. In terms of children’s gender, there was an almost equal split between boys (48%) and girls (52%).

**Table 2:** Participant sociodemographic and anthropometric characteristics (*N* = 466)

Continuous variables	Mean ± SD	Min—Max
Mothers’ age (y)	31.53 ± 4.75	19—43
Children’s age (mo)	11 ± 3.5	4—18
Nominal variables	*N*	Proportion %
Children’s gender		
Boys / girls	221 / 241	47.8 / 52.2
Ethnic background		
White	453	97.2
Black/Black British	1	0.2
Asian/Asian British	5	1.1
Mixed/other ethnic background	7	1.5
Location of residence		
England	322	70.2
Northern Ireland	71	15.5
Wales	37	8.1
Scotland	29	6.3
Marital status		
Single	24	5.2
Cohabiting	138	29.7
Married	299	64.3
Separated	4	0.9
Educational attainment		
Primary school	2	0.4
GCSE’s	26	5.6
A’ levels	85	18.2
Undergraduate	203	43.6
Postgraduate	150	32.2
Household income (£)		
< 10 k	16	3.5
10—20 k	32	7.1
20—30 k	61	13.5
30—40 k	85	18.8
40—60 k	257	57
Total number of children		
1 / 2 / 3 / 4 or more	326 / 112 / 22 / 6	70/ 24 / 4.7 / 1.3
Mothers’ BMI (kg/cm^2^)		
Underweight	8	2
Normal weight	189	47
Overweight	119	29.6
Obese	51	12.7
Obese II	23	5.7
Obese III	12	3
Children’s weight status		
Underweight	14	3.5
Normal weight	288	61.8
Overweight	54	11.6
Obese	39	

*GCSEs* General Certificate of Secondary Education, *Alevels* Advanced Level qualifications

Most infants in this study had been breastfed at some point (84%) and were offered complementary foods at 6 months in compliance with the Department of Health recommendation (70%). Just over half of the mothers (55%) initiated complementary feeding based on the NHS recommendation to introduce complementary foods at 6 months, and 57% of mothers scored highly (between 0.8 and 1) in the Complementary Feeding Utility Index. A preference for feeding homemade complementary foods over ready-made ones was reported. Table 3 present in greater detail sample’s feeding practices.

**Table 3:** Participant complementary feeding practices (*N* = 466)

Continuous variables	Mean ± SD	Min—Max
Breastfeeding duration (mo)	7.3 ± 5.2	0 – 18
Timing of introduction of solids (wk)	24.7 ± 2.8	8 – 30
CFUI score	0.8 ± 0.12	0.3 – 1
Nominal variables	*N*	Proportion % ^1^
Breastfeeding (at all)		
Yes / No	390 / 76	83.7 / 16.3
Timing of introduction of solids (categories)		
≤ 17 weeks (up to 4 mo)	18	3.9
18 – 25 weeks	121	26
≥ 26 weeks (6 mo and after)	326	70.1
Quintiles of CFUI score^2^		
Q1 / Q2 / Q3 / Q4 / Q5	0/3/29/188/246	0/0.6/6.2/40.3/52.9
Prompts for introducing solids		
Baby was interested in food	69	14.8
Baby was developmentally ready	106	22.7
I followed the NHS recommendation	258	55.4
Other^3^	33	7.1
Feeding commercial infant foods		
Yes / never	62 / 155	28.6 / 71.4

^1^Proportions are expressed as valid percentages due to missing cases for every variable

^2^CFUI scoring range was split into equal quintiles to show the distribution of diet quality scores across the sample

^3^Other, less frequently reported triggers for complementary feeding include: being encouraged by the health visitor, changes in baby’s weight, perceived hunger, disrupted sleep and mother’s desire to stop breastfeeding

In relation to feeding foods from specific food groups, 32% mothers reported offering three or more vegetables every day, and 27% and 37% offered one or two vegetables respectively; only 4% reported not offering any vegetables on a daily basis. With fruits, 40% of mothers offered three or more fruits a day, 33% offered two and 24% one fruit a day; 3% offered less frequently than every day. In relation to the consumption of sweetened drinks in this sample 81% of the participants didn’t offer any sweet drinks including fruit juice. From those who offered some type of drinks the most common was fruit squash (i.e. drink made from concentrated fruit syrup diluted in water) (10%) followed by fruit juice (7%); data on portion sizes or preparation method (diluted or not) was not available. A significant proportion of mothers (46%) reported not offering any high-fat, high-sugar snacks to their infants, whereas 20% offered one type per week, 19% two and 13% three. Savoury snacks (e.g. crisps and cheesy biscuits) were the most frequently offered food among those who reported giving foods from this category (46%) on a weekly basis.

### Associations with dietary quality

A multivariate linear regression model was used to examine independent associations between the covariates and dietary quality, as shown in Table 4. Both scales for parental feeding self-efficacy were significant explanatory variables of dietary quality after adjusting for participant age, marital status, education, household income and BMI. Specifically, every unit increase in scores of self-efficacy in promoting healthy foods was associated with obtaining CFUI scores that were higher by 0.018 (*P* = 0.030), and self-efficacy in restricting unhealthy foods was associated with CFUI scores higher by 0.026 (*P* = 0.002). Reliance on the NHS guidelines was also an independent predictor of diet quality in this model. Following the NHS recommendations as a prompt to introduce solids was associated with an increase in CFUI by 0.026 compared to being prompted by other cues (*P* = 0.034). There was no evidence of an association between dietary quality and parental food neophobia, perceived social support, postnatal depression or infant temperament based on the study data.

**Table 4:** Multivariate regression model with CFUI score as a dependent variable, (Adjusted R^2^ = 12.8%)

Predictive variables	Mean ± SD	Min—Max	B	95% CI	*P*^1^
Reliance on NHS recommendations			**0.026**	**0.002, 0.050**	**0.034**
Food neophobia (38)	19.01 ± 8.6	0—47	-0.001	-0.003, 0	0.160
Feeding self-efficacy (15)					
Self-efficacy in promoting healthy foods	4.15 ± 0.8	1—5	**0.018**	**0.002, 0.033**	**0.030**
Self-efficacy in limiting non-core foods	4.23 ± 0.75	1—5	**0.026**	**0.010, 0.043**	**0.002**
Social support (41)	4.34 ± 0.72	1.17—5	-0.011	-0.029, 0.008	0.261
Postnatal depression (43)	7.91 ± 4.97	0—26	-0.003	-0.005, 0	0.055
Infant temperament (45)					
Activity level ^2^	2.91 ± 0.87	1—5	-0.001	-0.015, 0.014	0.922
Distress to limitations ^2^	2.65 ± 0.90	1—5	0.004	-0.009, 0.018	0.534
Smiling & laughter ^2^	3.93 ± 0.69	1—5	0.003	-0.015, 0.021	0.750

^1^*P* values occurred after adjusting for participant age, marital status, education, household income and BMI

^2^Only three domains were selected out of the 14 domains of the original Infant Behaviour Questionnaire -Revised, as these have been associated with parental feeding practices in previous literature

*B* Unstandardised regression coefficient, *CI* Confidence interval for B

### Associations with adherence to recommended feeding practices

The ordinal regression model included only the explanatory variables that had fewer missing values than 50% of the sample. These were maternal feeding practices including the timing of introduction of solid foods, exposure to vegetables, to fruits, to protein sources, sweet drinks and confectionary and savoury snacks. Table 5 presents the regression coefficients (Estimate b) and *P* values after controlling for a number of demographic characteristics (as for total CFUI score).

**Table 5:** Multivariate regression model with individual CFUI items (ordinal variables) as dependent variables

Predictive variables ^1^	Timing of introducing solids		Exposure to vegetables		Exposure to fruits		Variety of protein-rich foods		Exposure to sweet drinks		Exposure to confectionary	
	*R*^2^ = 53.4%		*R*^2^ = 15.2%		*R*^2^ = 10.9%		*R*^2^ = 9.7%		*R*^2^ = 11.7%		*R*^2^ = 13.8%	
	Est (b)	*P*	Est (b)	*P*	Est (b)	*P*	Est (b)	*P*	Est (b)	*P*	Est (b)	*P*
Participant age	**-0.095**	**0.020**	0.021	0.426	0.013	0.724	-0.006	0.809	**0.076**	**0.042**	**0.056**	**0.032**
Maternal BMI	0.007	0.804	0.010	0.596	0.007	0.778	0.015	0.437	-0.016	0.529	**-0.041**	**0.027**
Food neophobia	-0.030	0.187	0.001	0.922	-0.005	0.772	-0.023	0.110	0.013	0.489	-0.006	0.666
Self-efficacy in promoting healthy foods	-0.086	0.696	**0.537**	** < 0.001**	0.126	0.481	0.165	0.250	0.108	0.567	0.083	0.550
Self-efficacy in limiting non-core foods	0.056	0.800	0.074	0.627	**0.418**	**0.027**	0.121	0.413	**0.522**	**0.006**	0.081	0.581
Social support	-0.342	0.180	0.009	0.960	-0.343	0.148	-0.005	0.978	0.046	0.835	0.091	0.582
Postnatal depression	-0.039	0.318	-0.003	0.916	-0.056	0.097	-0.017	0.506	0.013	0.708	-0.045	0.076
Activity level	-0.152	0.567	**0.344**	**0.009**	**0.460**	**0.009**	-0.014	0.916	-0.084	0.650	-0.024	0.856
Distress to limitations	**0.470**	**0.014**	-0.231	0.070	0.018	0.913	-0.009	0.940	-0.059	0.735	-0.046	0.710
Smiling & laughter	-0.173	0.453	0.320	0.051	0.183	0.364	0.106	0.523	0.074	0.737	-0.278	0.092
Reliance on NHS recommendations	**4.201**	** < 0.001**	0.032	0.884	0.146	0.601	0.328	0.136	0.348	0.241	-0.388	0.070

^1^ The model for every dependent variable was adjusted for marital status, education and household income

The regression model revealed that reliance on the NHS guidelines and high levels of infant distress to limitations were positively associated with adhering to the recommended timing of introduction of solids (*P* < 0.001, *P* = 0.014 respectively), whereas higher maternal age was adversely associated with it (*P* = 0.020). Maternal self-efficacy in feeding was positively associated with following a number of complementary feeding recommendations; mothers with higher self-efficacy in promoting healthy foods were more likely to offer vegetables three or more times daily (*P* < 0.001) and those with high self-efficacy in avoiding unhealthy foods were more likely to offer two or more fruits a day (*P* = 0.027) and to avoid giving sweet drinks (*P* = 0.006).

Infant temperament had an overall significant effect on the amount of vegetables and fruit offered daily in the adjusted model. Infants that scored highly in activity level were more likely to be given the recommended daily intake in fruits and vegetables (*P* = 0.009 for both). Maternal BMI was a significant predictor of children’s exposure to unhealthy snacks with every unit increase in BMI associated with decreased odds of adhering to the guideline on avoiding high-fat/-sugar snacks (*P* = 0.027). Additionally, maternal age was a significant determinant of the exposure to sweetened drinks and exposure to confectionary and savoury snacks and older mothers were more likely to limit the consumption of these foods (*P* = 0.042 and *P* = 0.032 respectively). Food neophobia, perceived social support and postnatal depression were not predictors of compliance with the complementary feeding recommendations in the adjusted model.

### Associations with child weight status

The regression coefficients and significant tests for each of the independent variables in the model are presented in Table 6. Breastfeeding duration, exposure to vegetables, to fruits, to animal foods high in protein and to sweet drinks were significantly associated with child weight status in the sample. Specifically, children that were breastfed for longer were less likely to be heavier (*P* = 0.009). Children who were offered vegetables once per day or less frequently than daily were less likely to be in a higher weight category compared with those who ate three or more vegetables (*P* = 0.008 and *P* = 0.002 respectively). Offering children one or no fruit daily was associated with being at an increased weight status when compared with offering two fruits (*P* = 0.026 and *P* = 0.047 respectively). Having three foods from the group of meat/fish/egg per week was associated with being in a lower weight category than having two of these foods per week (*P* = 0.034). Weekly intake of one sweet drink was related to greater risk of increased weight when compared to zero intake (*P* = 0.006).

Additionally, the age of introduction of lumpy textures in food and responsive feeding were significantly associated with children’s weight in this sample. Children that had lumpy foods at some point between 6 and 8 months were less likely to be in a heavier weight category compared to those fed lumps earlier or later (*P* < 0.001 and *P* = 0.005). Children that were fed on demand, i.e. when they showed hunger, were less likely to be heavier compared to children fed on schedule (*P* = 0.033). No association was observed between children’s weight status and consumption of confectionary and commercial foods in the adjusted model.

**Table 6:** Multivariate regression with children weight status (ordinal variable) as dependent variable (Nagelkerke R^2^ = 63%)

Predictive variables	Est (b)	*P*
Breastfeeding duration	**-2.681**	**0.009**
Exposure to vegetables		
0 per day	**-5.111**	**0.002**
1 per day	**-2.831**	**0.008**
2 per day	-1.295	0.143
3 or more per day	-	-
Exposure to fruit		
0 per day	**5.119**	**0.026**
1 per day	**1.631**	**0.047**
2 or more per day	-	-
Variety in protein sources		
0 types per week	0.467	0.709
1 type per week	1.530	0.225
2 types per week	**1.460**	**0.034**
3 types per week	-	-
Exposure to sweet drinks		
2 type	1.912	0.150
1 types	**2.743**	**0.006**
0 types	-	-
Exposure to confectionary and savoury snacks		
4 types	0.575	0.709
3 type	-2.041	0.061
2 types	-0.536	0.601
1 types	0.064	0.943
0 types	-	-
Consumption of manufactured baby foods	0.159	0.833
Timing of introduction of lumpy foods		
Late (> 8mo)	**7.190**	**0.005**
Early (4-6mo)	**7.397**	** < 0.001**
On time (6-8mo)	-	-
Responsive feeding		
No—fed on schedule	**2.584**	**0.033**
Sometimes fed on demand	-1.408	0.079
Yes—fed on demand	-	-

*P* values occurred after adjusting for baby age, maternal education, household income and maternal BMI

## Discussion

This study used cross-sectional data from mothers living in the UK to assess the dietary quality of their children during the complementary feeding period using the Complementary Feeding Utility Index. It also tested the association between dietary quality and a number of maternal and infant characteristics, as well as the association between maternal compliance with specific feeding guidelines and child weight status. High dietary quality was predicted by reliance on the NHS recommendation to introduce solid foods and by maternal self-efficacy in promoting healthy foods and limiting non-recommended foods. In relation to child weight status, our findings show that being in a heavier weight category was positively associated with frequent exposure to sweet drinks, high consumption of vegetables and delayed introduction of lumpy food textures. Additionally, longer breastfeeding duration, responsive feeding, high variety in animal protein sources and high fruit intake were inversely associated with being in a heavier weight category. To the authors’ knowledge, this study is the first to assess the quality of complementary feeding diets in a UK-wide sample using a validated instrument which considers multiple aspects of diet. It is also the first study to explore the association between diet quality and maternal and child behavioural, psychological and sociological aspects.

### Findings regarding dietary quality

The reported dietary quality was high for the sample with the average CFUI score being 0.8, which was higher than the mean CFUI score observed in a large UK cohort conducted in 1990 by Golley et al. (0.5) [34]. The present analysis takes place almost two decades after Golley’s analysis, since which great advances in nutrition research have been achieved. Hence, differences in CFUI scores hopefully reflect better maternal knowledge of the recommended and non-recommended feeding practices.

The Complementary Feeding Utility Index provided a validated measure of dietary quality for the target population and hence, was considered appropriate for this study. However, its development entailed arbitrary choices, similar to other dietary quality indices [25]. As the authors of the index point out, a total CFUI score is obtained by linear aggregation of the individual component scores which assumes that all individual components are interchangeable [34]. Additionally, CFUI’s ability to identify cases of low food diversity is limited. Finally, a large number of missing data was observed in this study for some of its individual components and, as the relevant questions were relatively early in this survey, this may reveal participants’ uncertainty on how to answer them (as opposed to respondent fatigue). These questions could be omitted or replaced in future measures of dietary quality in early years.

In this study dietary quality during complementary feeding was predicted by mothers’ self-efficacy in promoting healthy foods and in restricting non-recommended snacks. At the same time, mothers who were prompted by the NHS guidelines to introduce solid foods were more likely to provide their children with a diet that is compliant to the complementary feeding guidelines. In Golley et al.’s study total CFUI scores were associated with demographic factors such as maternal age, education, marital status and BMI, whereas in this study, none of these characteristics were identified as significant predictors of dietary quality. Mothers in this study however, were mostly white, married, well-educated and from wealthy households which may explain these contrasting findings.

### Findings regarding maternal self-efficacy

Study findings revealed high levels of maternal feeding self-efficacy demonstrating that mothers in the sample perceived themselves to have good ability to encourage their children to eat a balanced diet and to restrict children’s intake in unhealthy snacks. Apart from a positive relationship with overall dietary quality, study results showed that maternal feeding self-efficacy was also significantly associated with maternal complementary feeding practices; there was a positive correlation with frequent vegetable exposure and negative correlation with frequent exposure to sweet drinks. The present findings validate the existing literature, as several studies have found that high feeding self-efficacy is a significant predictor of eating patterns higher in fruit and vegetables and low in snack foods in infants and young children [12, 14–16]. Present findings also add to the body of evidence supporting that mothers who perceive themselves to have good feeding skills can effectively foster a healthy diet for their children.

### Findings regarding reliance on the official complementary feeding guidelines

The association between relying on the NHS guidelines and the level of dietary quality introduces a novel insight into factors that can determine the quality of complementary diet, as this relationship has not been explored so far. It is possible that increased trust in the guidance on timing of complementary feeding may be also correlated with good awareness of the overall complementary feeding recommendations, which can in turn encourage positive feeding practices to ensure food variety and avoid unsuitable foods.

### Findings regarding infant temperament

In this study, aspects of children’s temperament, measured by the Infant Behaviour Questionnaire Revised, were correlated with several feeding practices and participants whose children were more physically active provided them with more fruit and vegetables. Considering the existing literature and present findings, there is mixed evidence regarding the role that infant temperament plays in shaping maternal feeding patterns. In a Norwegian cohort, infants who were more surgent (social and active) were more likely to be given sweetened drinks [49], whereas in an Australian cross-sectional study, mothers of infants with a more difficult temperament were more likely to use food to sooth [50]. More studies are required to explore the relationship between different dimensions of child temperament and complementary feeding practices.

### Findings regarding maternal age and BMI

Participants’ age and adiposity were not associated with overall children’s diet quality, but predicted adherence to some feeding recommendations. Older mothers avoided offering sweet drinks and unhealthy snacks, which may reflect good awareness of foods to avoid during complementary feeding. However, the narrow age range of mothers (most were between 27–37 years old) needs to be pointed out; a wider range would facilitate better exploration of how maternal age correlates to feeding patterns. Furthermore, maternal BMI had a positive relationship with frequent exposure to confectionary and savoury snacks such as chocolate bars, crisps and sweets. A previous UK study supports that mothers living with excess weight are less likely to restrict the consumption of unhealthy snacks compared with mothers of healthy weight [51]. Hence, present findings highlight the importance of weight management support provided to women before, during and after pregnancy to influence their own health and behaviours and that of the wider family.

### Findings regarding the association between feeding practices and infant weight

There was a high prevalence of increased adiposity in the sample with one in five children being overweight or obese. It is not certain how this proportion compares to national UK data, as the Diet and Nutrition Survey for Infants and Young Children reports only the proportion of children that are above the 50th centile of the weight curve [5], whilst for this study children were classified overweight and obese if they were above the 91st and 98th centile respectively, as per the RCPCH guidance [36]. Shorter breastfeeding duration was a significant determinant of increased body weight adjusted for age and gender, which is consistent with wider literature [52–55].

Having excess weight during complementary feeding was also predicted by a feeding pattern characterised by frequent consumption of sweetened drinks and low variety of animal protein sources. Frequent exposure to drinks containing sugar has been formerly identified as a risk factor of child overweight, although findings are not consistent across studies [56]. Regarding the role of protein-rich foods in relation to BMI, mixed findings have emerged from research. Even though total intake of animal protein is directly linked with growth in the first year [57, 58], an investigation of 2-year-olds in the UK observed that a dietary pattern characterised by greater diversity in protein sources may confer a lower risk of excess weight gain [59], which is in agreement with present findings. It is possible that greater diversity in protein sources is a marker of overall greater diet diversity and higher adherence to dietary recommendations with benefits for infant’s weight. Further research on the association between dietary protein diversity and body weight in infancy and later childhood is required.

Higher intake of vegetables but lower intake of fruits were associated with excess weight in the studied age group. The association with fruit has been previously reported, but with vegetables is surprising, since high vegetable intake has been associated with lower risk of overweight [60]. In this study however, findings may reflect that children who ate vegetables ate generally more and therefore, had a more accelerated growth pattern. This however, remains unknown, as there were no information regarding children’s overall dietary intake. Additionally, these findings suggest that a transition from pureed to lumpy foods between 6 and 8 months may help maintain a healthy weight. The importance of an appropriate texture introduction during complementary feeding for food acceptance has been highlighted in previous studies [4, 61], but a direct link with children’s growth pattern has not been fully explored yet.

Finally, mothers who fed their children based on their hunger cues were less likely to have a heavy child in this study. This finding is in alignment with previous evidence suggesting that responsive feeding can lead to a healthy weight gain in children from birth to 24 months [62]. It also highlights the importance of appropriately using child’s appetite cues to guide feeding.

Overall dietary quality in this study didn’t seem to correlate to weight status; contrary to an observation by Rios et al. that infants and toddlers who had diets of poor quality were more likely to have excessive weight [31]. The contrasting findings may be due to the fact that the majority of infants in this study had a high dietary quality. Further research work is warranted to clarify how early dietary patterns contribute to the development of childhood obesity.

### Limitations

This paper draws on cross-sectional data and it is therefore, impossible to establish any associations between maternal feeding practices and children’s eating behaviours or weight status in the long term. Nonetheless, evidence supports that food preferences are established during complementary feeding [63] and rapid weight gain during the first year may result to overweight later in life [64]. Therefore, awareness of early determinants of good eating habits and of a healthy growth pattern, as identified in this paper, is valuable.

Although the online design enabled us to collect data from a geographically disperse population, it also meant that the sample was self-selected and therefore, results are subject to selection bias. Mothers were in majority well-educated, affluent and white, whereas ethnic minority groups were underrepresented in this study; comprising a 3% of the sample contrary to the 15–25% of the UK population [65–67]. The low demographic diversity in the sample limits the generalisability of the results and may be responsible for the lack of associations between maternal demographics and feeding practices. Future studies need to consider sampling approaches targeted towards different ethnicities and low-income households. In addition, slightly under three quarters of the sample introduced solids from 6 months, which is three times higher than expected based on latest UK data [5]. Although it is possible that adherence to the recommended timing of complementary feeding has increased over the years, the proportion of mothers introducing solids at 6 months in this study is still much greater than in the UK population.

When completing the survey, participants needed to recall the timing of introducing solid foods and lumpy food textures which increased the risk of introducing recall bias. However, these two transitions are important nutritional milestones in a child’s life and it is therefore, expected that parents would remember them with a relative accuracy. All other questions were pertinent to parents’ current feeding practices. Finally, the self-reported nature of the survey also increases the risk of social desirability bias, whereby mothers might have reported children’s ‘desired intake’ as opposed to the ‘true intake’ [68].

## Conclusion

This study provides an evaluation of dietary quality in a UK sample of children in the complementary feeding age by using the Complementary Feeding Utility Index. It also explored the strength of the relationship between dietary quality and a number of maternal and infant attributes, as well as the relationship between maternal adherence to feeding guidelines and infant weight status. Maternal reliance on the official 6-month recommendation and maternal feeding self-efficacy were associated with high dietary quality during complementary feeding. The results also suggest that frequent exposure to sweet drinks, delayed introduction of lumpy foods, low intake of fruits, low variety of animal protein sources, non-responsive feeding approaches and discontinuation of breastfeeding increase children’s risk of excess weight.

This paper offers a number of important considerations for professionals involved in complementary feeding education. Mothers who have a good awareness of the complementary feeding recommendations can establish better feeding patterns and therefore increasing awareness of the recommendations should be an integral part of the support offered to young parents. Future research should explore methods to increase parental feeding self-efficacy; these methods then need to be incorporated in interventions aiming to improve complementary feeding behaviours.

## Supplementary Information

**Additional file 1.** Survey questionnaire: final questionnaire as completed by participants.

**Additional file 2.**  Survey dataset: data collected from participants included in the statistical analysis.

**Additional file 3.** Supplementary table - Infant Behaviour Questionnaire Revised – Short Form (IBQ-R): scale used to assess infant temperament (only 3 domains of the scale included).

## Data Availability

The datasets used and/or analysed during the current study are included in the supplementary information file (Additional file 2).
