# Supplementary material for: An online survey of dietary quality during complementary feeding; associations with maternal feeding self-efficacy and adherence to dietary recommendations
Source: BMC Nutr. 2022 Sep 9;8:100. doi: 10.1186/s40795-022-00595-8 (PMC9461111; doi:10.1186/s40795-022-00595-8)

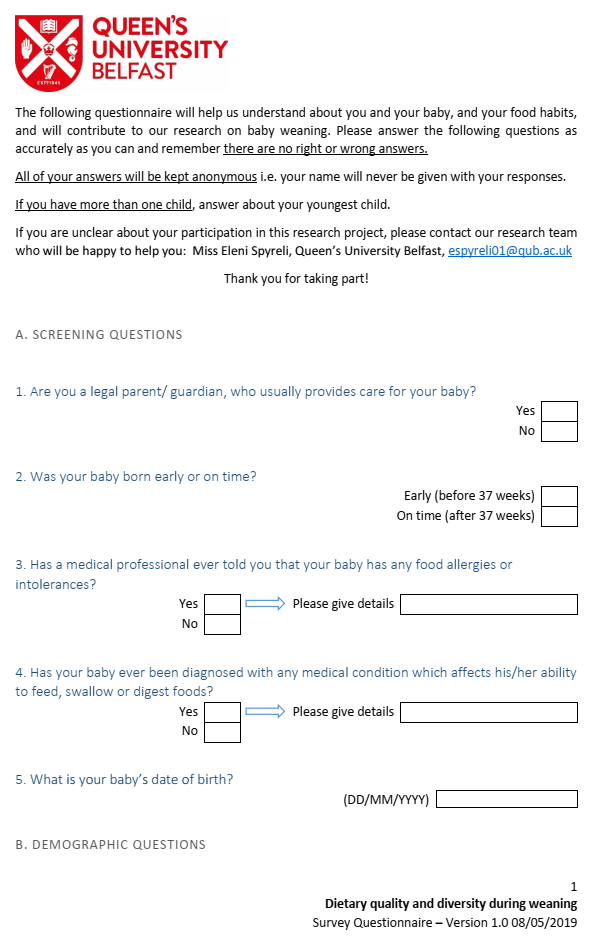


Survey questionnaire

Survey questionnaire (continued)

(Continued)


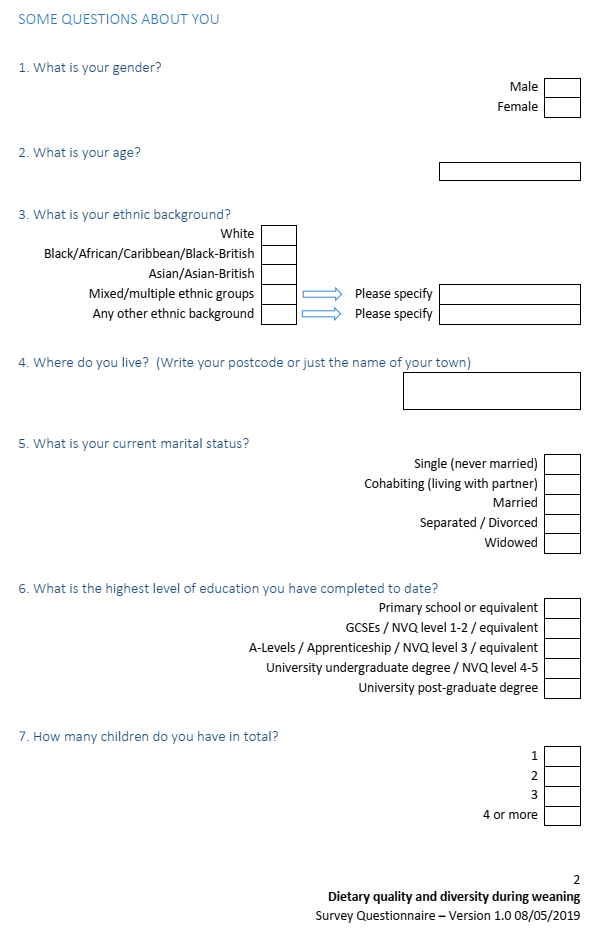


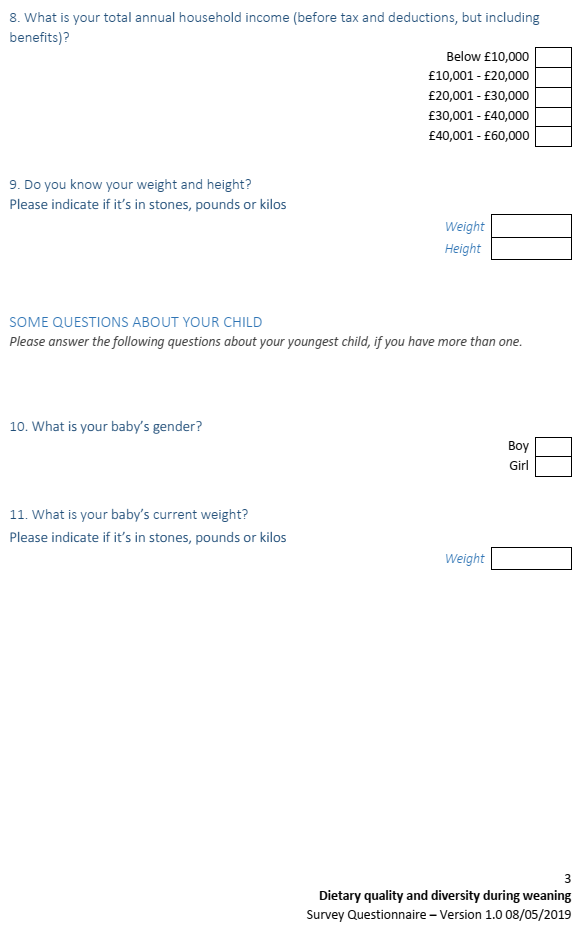


Survey questionnaire (continued)

(Continued)


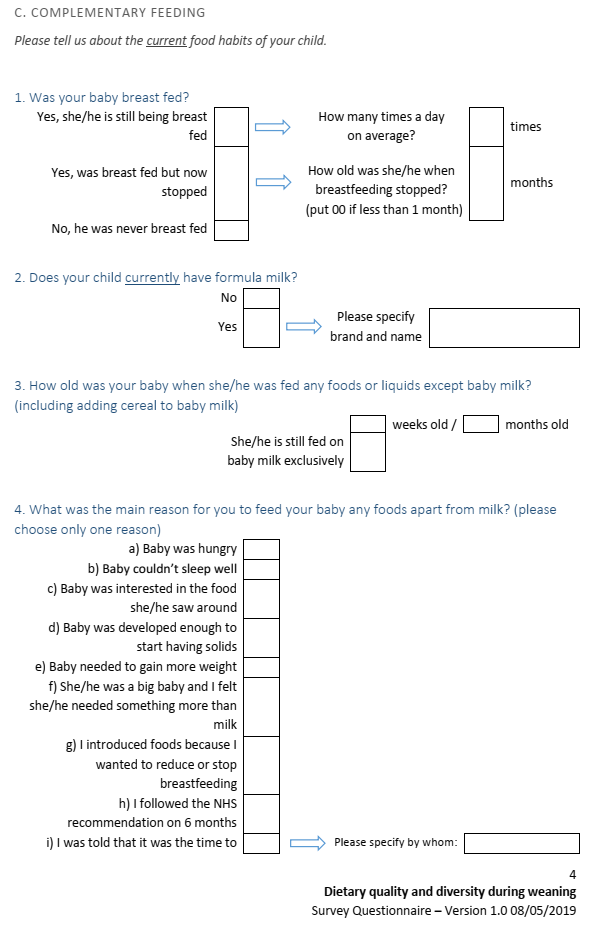


Survey questionnaire (continued)

(Continued)


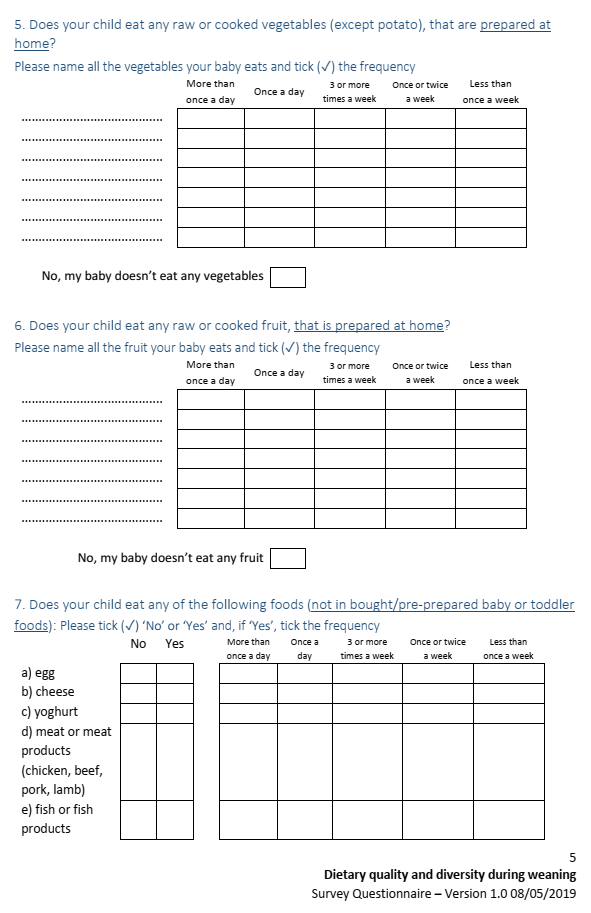


Survey questionnaire (continued)

(Continued)

Survey questionnaire (continued)

(Continued)


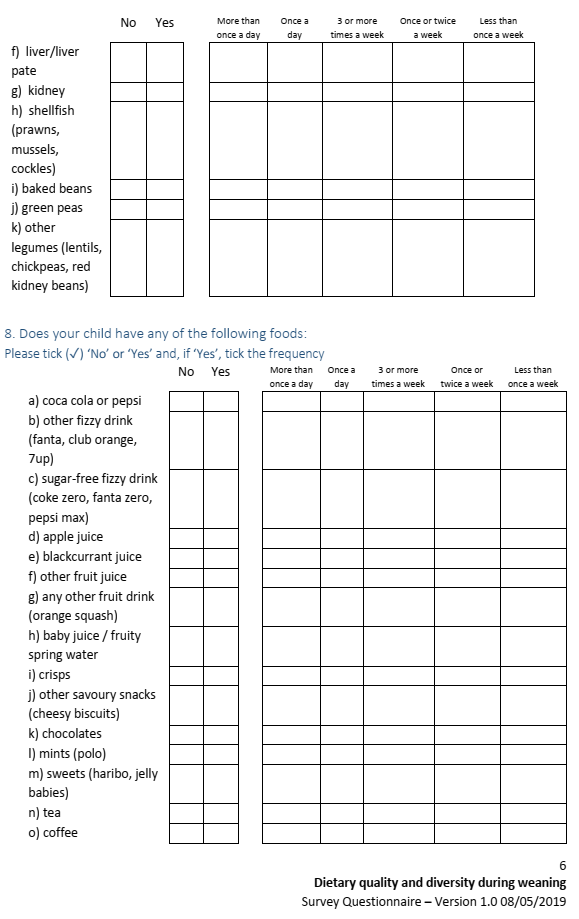


Survey questionnaire (continued)

(Continued)


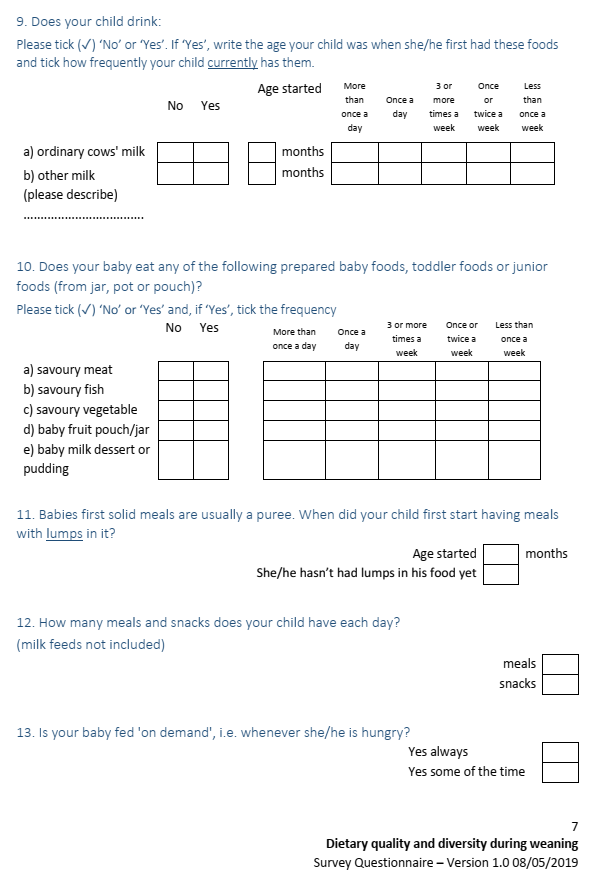


Survey questionnaire (continued)

(Continued)


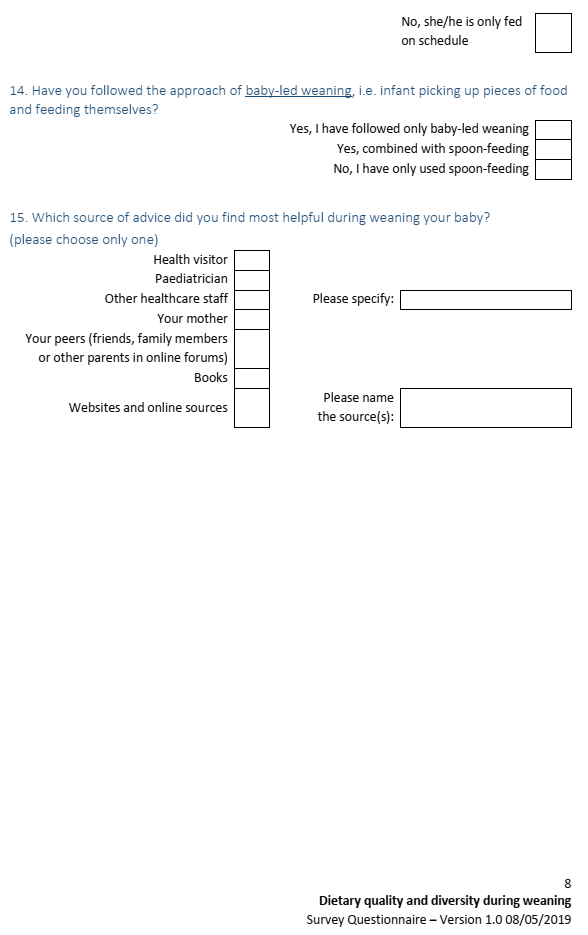


Survey questionnaire (continued)

(Continued)


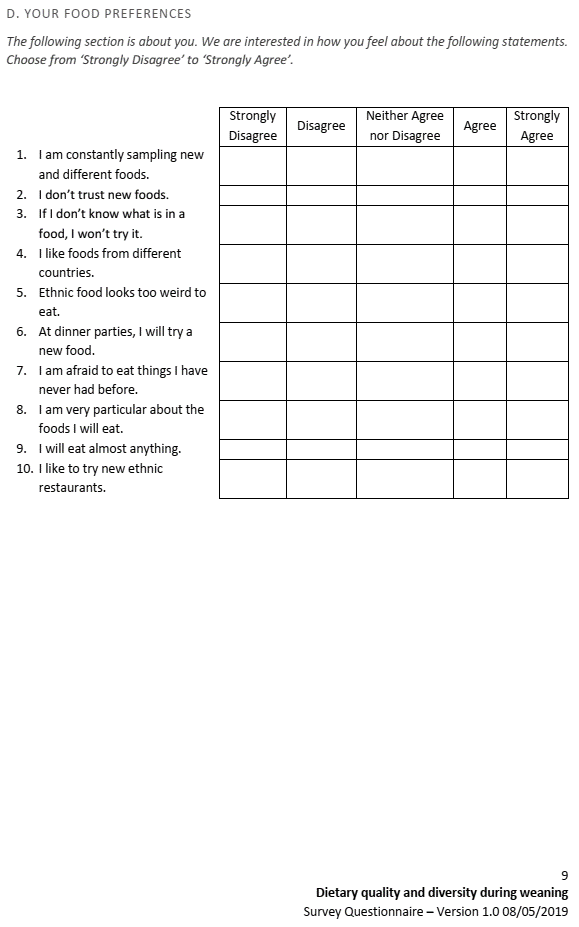


Survey questionnaire (continued)

(Continued)


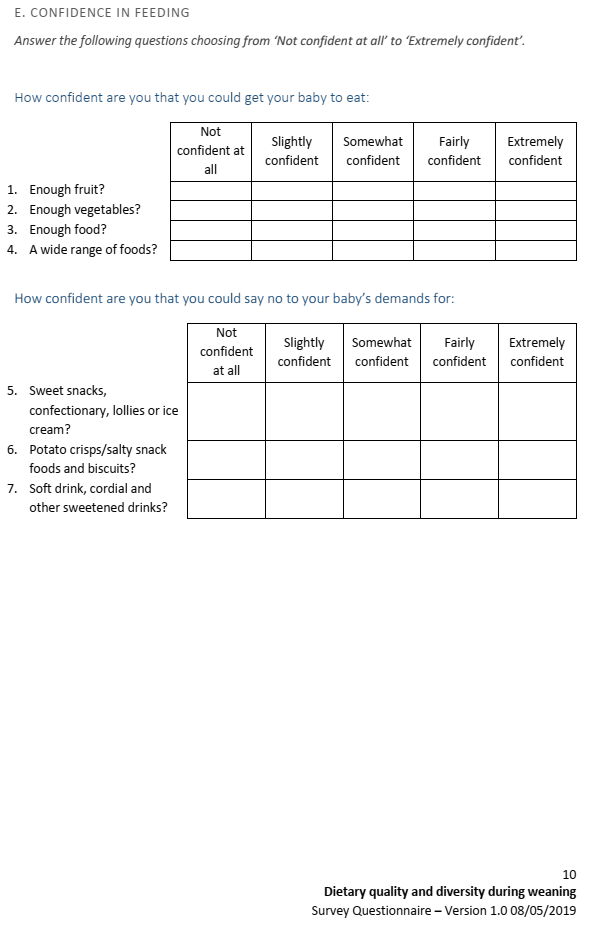


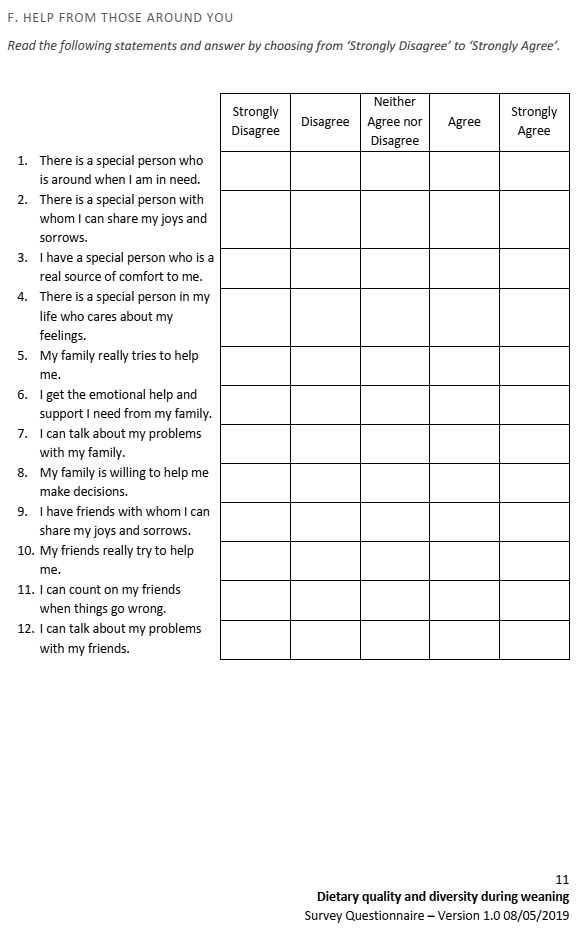


Survey questionnaire (continued)

(Continued)


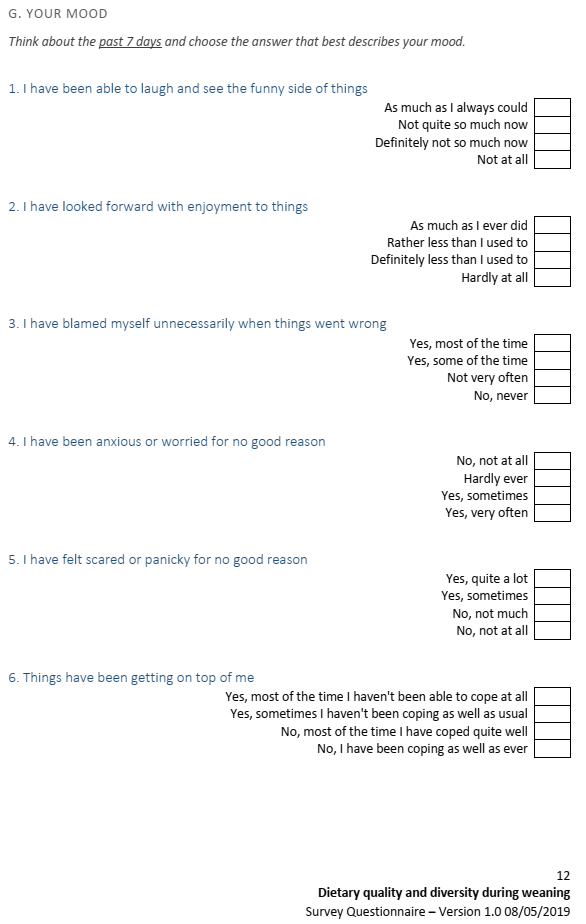


Survey questionnaire (continued)

(Continued)


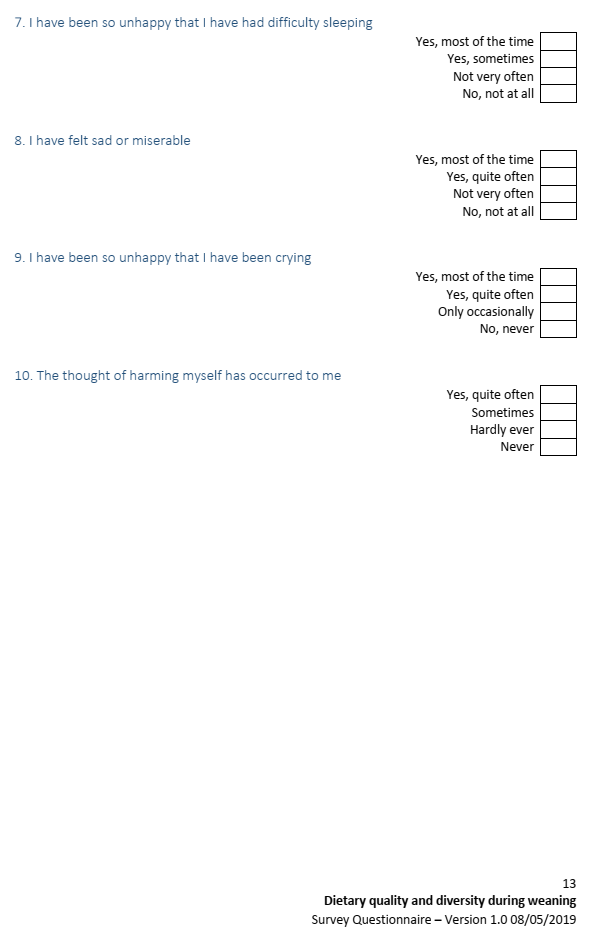


Survey questionnaire (continued)

(Continued)


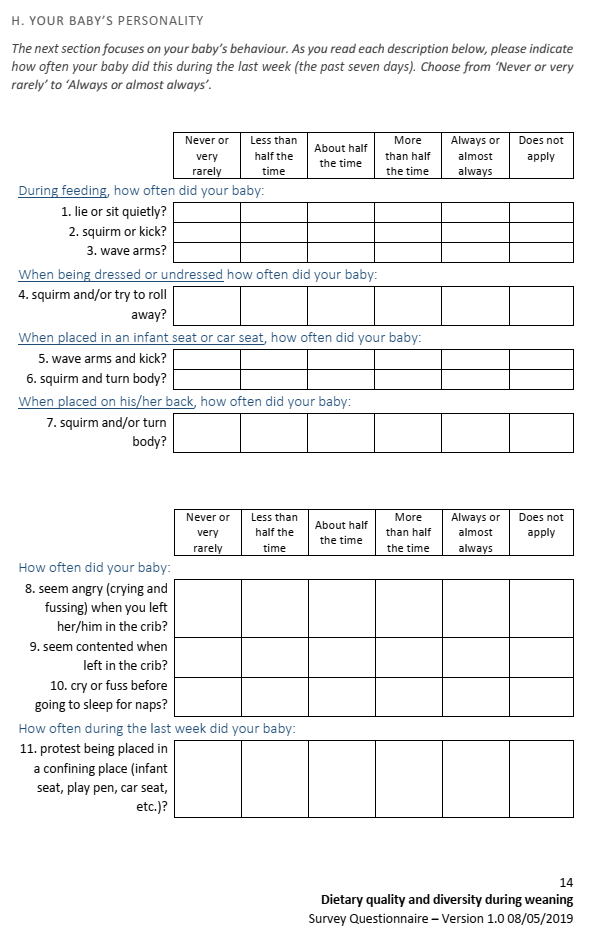


Survey questionnaire (continued)

(Continued)

Survey questionnaire (continued)

(Continued)


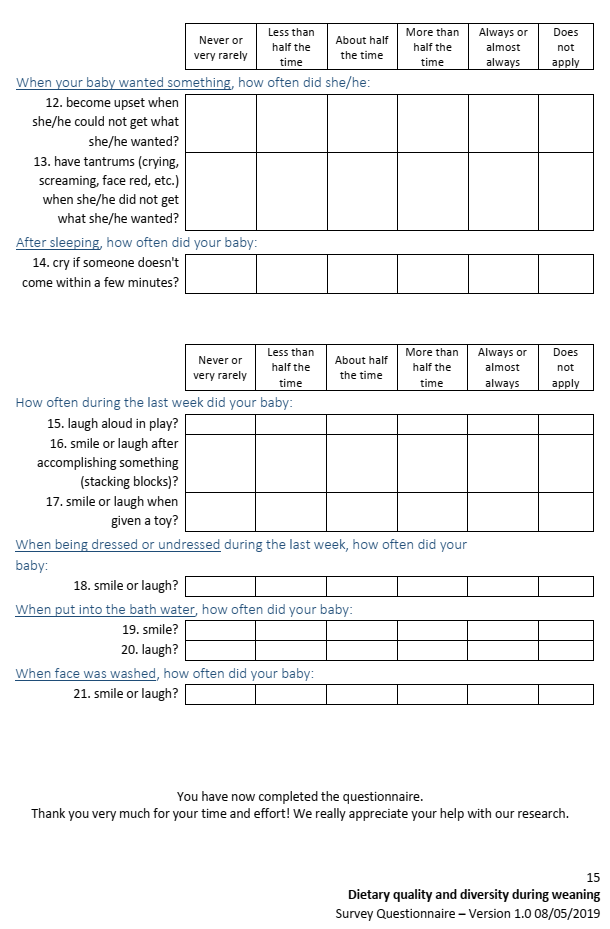

Supplement: Supplementary file 1 — Additional file 1. Survey questionnaire: final questionnaire as completed by participants. [file 40795_2022_595_MOESM1_ESM.docx]
