# Supplementary material for: An online survey of dietary quality during complementary feeding; associations with maternal feeding self-efficacy and adherence to dietary recommendations
Source: BMC Nutr. 2022 Sep 9;8:100. doi: 10.1186/s40795-022-00595-8 (PMC9461111; doi:10.1186/s40795-022-00595-8)
Supplement: Supplementary file 3 — Additional file 3. Supplementary table - Infant Behaviour Questionnaire Revised – Short Form (IBQ-R): scale used to assess infant temperament (only 3 domains of the scale included). [file 40795_2022_595_MOESM3_ESM.docx]

Infant Behaviour Questionnaire Revised – Short Form (IBQ-R) for the domains: “Activity Level”, “Distress to limitations” and “Smiling and Laughter”, as detailed by Putnam et al (45)

| Activity level | Never or very rarely | Less than half the time | About half the time | More than half the time | Always or almost always | | | Does not apply | | |
| --- | --- | --- | --- | --- | --- | --- | --- | --- | --- | --- |
| During feeding, how often did your baby: | | | | | | | |  | | |
| 1. lie or sit quietly? |  |  |  |  |  | | |  | | |
| 2. squirm or kick? |  |  |  |  |  | | |  | | |
| 3. wave arms? |  |  |  |  |  | | |  | | |
| When being dressed or undressed how often did your baby: | | | | | | |  | | |  |
| 4. squirm and/or try to roll away? |  |  |  |  |  | | |  | | |
| When placed in an infant seat or car seat, how often did your baby: | | | | | |  | | |  |  |
| 5. wave arms and kick? |  |  |  |  |  | | |  | | |
| 6. squirm and turn body? |  |  |  |  |  | | |  | | |
| When placed on his/her back, how often did your baby: | | | | | |  | | |  |  |
| 7. squirm and/or turn body? |  |  |  |  |  | | |  | | |

| Distress to limitations | Never or very rarely | Less than half the time | About half the time | More than half the time | Always or almost always | | | Does not apply | | |
| --- | --- | --- | --- | --- | --- | --- | --- | --- | --- | --- |
| How often did your baby: | | | | | |  | | |  |  |
| 8. seem angry (crying and fussing) when you left her/him in the crib? |  |  |  |  |  | | |  | | |
| 9. seem contented when left in the crib? |  |  |  |  |  | | |  | | |
| 10. cry or fuss before going to sleep for naps? |  |  |  |  |  | | |  | | |
| How often during the last week did your baby: | | | | | | |  | | |  |
| 11. protest being placed in a confining place (infant seat, play pen, car seat, etc.)? |  |  |  |  |  | | |  | | |

| \|  \| Never or very rarely \| Less than half the time \| About half the time \| More than half the time \| Always or almost always \| Does not apply \| \| --- \| --- \| --- \| --- \| --- \| --- \| --- \| \| When your baby wanted something, how often did she/he: \| \| \| \| \|  \| \| 12. become upset when she/he could not get what she/he wanted? \|  \|  \|  \|  \|  \|  \| \| 13. have tantrums (crying, screaming, face red, etc.) when she/he did not get what she/he wanted? \|  \|  \|  \|  \|  \|  \| \| After sleeping, how often did your baby: \| \| \| \| \|  \| \| 14. cry if someone doesn't come within a few minutes? \|  \|  \|  \|  \|  \|  \|  \| Smiling and laughter \| Never or very rarely \| Less than half the time \| About half the time \| More than half the time \| Always or almost always \| \| \| \| Does not apply \| \| \| \| --- \| --- \| --- \| --- \| --- \| --- \| --- \| --- \| --- \| --- \| --- \| --- \| \| How often during the last week did your baby: \| \| \| \| \|  \| \| \| \| \| 15. laugh aloud in play? \|  \|  \|  \|  \|  \| \| \| \|  \| \| \| \| 16. smile or laugh after accomplishing something  (stacking blocks)? \|  \|  \|  \|  \|  \| \| \| \|  \| \| \| \| 17. smile or laugh when given a toy? \|  \|  \|  \|  \|  \| \| \| \|  \| \| \| \| When being dressed or undressed during the last week, how often did your baby: \| \| \| \| \| \|  \| \| \| \| \| 18. smile or laugh? \|  \|  \|  \|  \|  \| \| \| \|  \| \| \| \| When put into the bath water, how often did your baby: \| \| \| \| \| \| \| \|  \| \| \| \| 19. smile? \|  \|  \|  \|  \|  \| \| \| \|  \| \| \| \| 20. laugh? \|  \|  \|  \|  \|  \| \| \| \|  \| \| \| \| When face was washed, how often did your baby: \| \| \| \| \| \| \|  \| \| \| \| \| \| 21. smile or laugh? \|  \|  \|  \|  \|  \| \| \| \|  \| \| \| |
| --- | --- | --- | --- | --- | --- | --- | --- | --- | --- | --- | --- | --- | --- | --- | --- | --- | --- | --- | --- | --- | --- | --- | --- | --- | --- | --- | --- | --- | --- | --- | --- | --- | --- | --- | --- | --- | --- | --- | --- | --- | --- | --- | --- | --- | --- | --- | --- | --- | --- | --- | --- | --- | --- | --- | --- | --- | --- | --- | --- | --- | --- | --- | --- | --- | --- | --- | --- | --- | --- | --- | --- | --- | --- | --- | --- | --- | --- | --- | --- | --- | --- | --- | --- | --- | --- | --- | --- | --- | --- | --- | --- | --- | --- | --- | --- | --- | --- | --- | --- | --- | --- | --- | --- | --- | --- | --- | --- | --- | --- | --- | --- | --- | --- | --- | --- | --- | --- | --- | --- | --- | --- | --- | --- | --- | --- | --- | --- | --- | --- | --- | --- | --- | --- | --- | --- | --- | --- | --- | --- | --- | --- | --- | --- | --- | --- | --- | --- | --- | --- | --- | --- | --- | --- | --- | --- | --- | --- | --- | --- | --- | --- | --- | --- | --- | --- | --- | --- | --- | --- | --- | --- | --- | --- | --- | --- | --- | --- | --- |
